# Neonatal epididymo-orchitis caused by Pseudomonas aeruginosa: a case report

**DOI:** 10.1186/1757-1626-3-44

**Published:** 2010-02-02

**Authors:** Meryem Kabiri, Amina Barkat, Houda El Ajaje, Nazek Allali, Rachida Dafiri, Naima Lamdouar-Bouazzaoui

**Affiliations:** 1 National centre of Neonatology and Nutrition Children's Hospital, Rabat 10100, Morocco; 2 Department of Paediatrics Radiology, Children's Hospital, Rabat 10100, Morocco

## Abstract

Epididymitis and epididymo-orchitis are an uncommon causes of acute testicular pain in neonatal boys, epididymo-orchitis is infection or inflammation of epididymis and testis it's may be associated with urinary tract infections or reflux of urine predisposed by an underlying vasal anomaly. Pediatricians should examine the testicles meticulously after a baby is born.

We report a 7 day-old boy with urinary malformations (ureteral duplication, ureterocel and right hydro-ureteronephrosis) who presented with acute scrotum. The ultrasonography exploration of the testis showed findings consistent with epididymo-orchitis, confirmed by the needle scrotal aspiration of the pus. Further radiological investigations of urinary tract showed the multiples malformations. Epididymo-orchitis should be suspected initially with abnormal physical signs and laboratory findings. Prompt prescription of antibiotics is mandatory, and appropriate therapeutic measures (antibiotics) should be undertaken to prevent recurrences and sequelae.

## Introduction

Neonatal testicular torsion and epididymo-orchitis are confusing and very difficult for medical doctors to diagnostic. Scrotal swelling in newborn is not rare and more diagnosis must be distinguished like: hydrocele, testicular torsion, orchitis, orchi-epididymitis, inguinal hernia, scrotal hematoma and tumors. The emergency cause is testicular torsion that requires surgical intervention but epididymo-orchitis is treated medically.

## Case presentation

**Figure 1:**
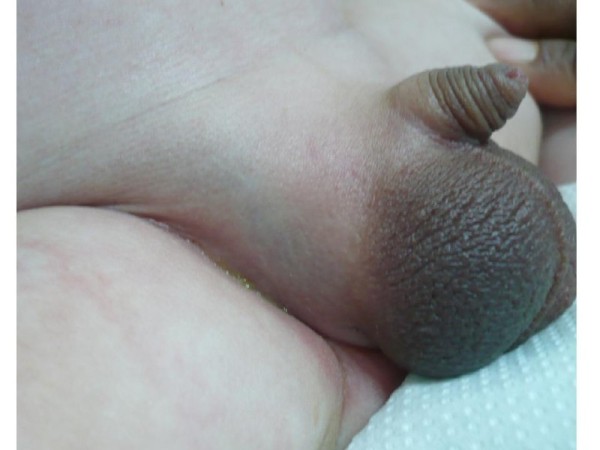
**Swelling and erythematous right hemiscrotum**.

A newborn Moroccan male was admitted in the intensive care unit at 2 hours with neonatal asphyxia signs. The infectious laboratory exams were normal. He was intubeted with good evolution. At 7 days the patient was presented the signs of infection: was tachycardic, his body temperature was 38.5°C. The clinical examination revealed a firm, dolorous, and erythematous right hemiscrotum (Figure 1). The laboratory exams revealed a WBC count of 28/10^9^ml^-1^, C reactive protein of 71 mg/l, the blood culture was positive (presence of Pseudomonas aeruginosa). Cultures of the cerebrospinal fluid and Urine were negative.

The color Doppler echography of testicular revealed increased vascular flow and heterogeneous aspects of the testis and epididymis with scrotal infusion. The needle punction aspirated the pus and the culture finds pseudomonas aeruginosa.

**Figure 2:**
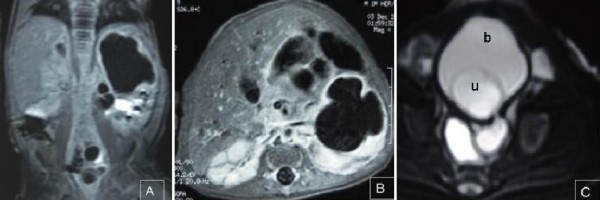
**Uro-Magnetic Resonance Imaging showed a left ureteral duplication (A), hydro-ureternephrosis (A, B) and ureterocel (C), (b: bladder, u: ureterocel)**.

The abdominal ultrasonography revealed a left hydro-ureteronephrosis with ureteral duplication and ureterocel. This malformative association was confirmed by abdominal magnetic resonance imaging (MRI) with Gadolium contrast (Figure 2).

The antibiotics were started with the imepinime 30 mg/kg/j for 10 days and Amikacine 15 mg/kg/j for 3 days. The evolution was good and ultrasonographic control of the testis was normal.

## Discussion

Epididymo-orchitis (EO) is a rare affection in the neonatal period. It's should be distinguished from testicular torsion to avoid unnecessary surgical exploration. Testicular torsion requires surgical intervention, but EO is managed medically [1].

The physical examination revealed a swollen testis, pain and fever, but these signs are not specific for EO [1,2].

The Color Doppler ultrasonography of the scrotum is capable to confirm the diagnosis and eliminated the testicular torsion. In EO, Doppler ultrasonography objective the increased vascular flow and the inhomogeneous echogenicity of the epididymis and the testis [3].

Retrograde passage of sterile or infected urine along the patent vas deferens is the most frequent cause of EO. Bloodstream infection is also reported [2,4]. EO is usually occurred in patient with predisposing anatomical abnormality. And All patients with EO should have an ultrasound examination of the abdominal and pelvic in order to determinate the anatomical abnormality of the urinary tract, such as an ectopic uretere, ureteral duplication or others malformations [2].

During the neonatal period, pseudomonas aeruginosa is responsible for nosocomial infection and it's difficult to treat. The acute epididymo-orchitis caused by pseudomonas aeruginosa is unusual and the clinical manifestations are similar to those caused by other micro organisms. *Escherichia coli *is an important gram-negative bacteria causing diverse neonatal infections and is also the common bacteria causing epididymo-orchitis from an ascending route. In our case it is the pseudomonas which is accused because it was about a nosocomiale infection. The choice of the imepineme is motivated by the bacterial ecology of service constituted by pseudomonas resisting to C3G, sensitive to the imipinème. Fact confirmed in the antibiogram.

Epididymo-orchitis is a rare affection in the neonatal period. After eliminate the torsion of the testicle, when EO is suspected, laboratory exams must done (urine exam, blood culture, and culture of the pus, and prompt antibiotics is prescribed to avoid serious sequelae.

## Abbreviations

EO: Epididymo-orchitis.

## Consent

Written informed consent was obtained from the patient's parents for publication of this case report and accompanying images. A copy of the written consent is available for review by the Editor-in-Chief of this journal.

## Competing interests

The authors declare that they have no competing interests.

## Authors' contributions

AB was major contributor in writing the manuscript and interpreted the data regarding the treatment and outcomes. NA, RD gathered and analysed the data regarding the radiological imaging. HEJ was also a major contributor in writing the manuscript. NLB was the neonatologist consultant in charge of the patient and provided the intellectual basis for the report; in addition, she was a major contributor to the discussion. All authors read and approved the final manuscript.

## References

[B1] Chiang MC Wang TM Fu RH Chu SM Chou YH Early-onset Escherichia Coli sepsis presenting as acute scrotum in preterm infant Urology 2005 65 389 10.1016/j.urology.2004.09.003 15708069

[B2] O'Brien M Chandran H The acute scrotum in childhood Surgery 2008 22 255 257

[B3] Atkinson GO Jr Patrick LE Ball TI Jr Stephenson CA Broecker BH Woodard JR The normal and abnormal scrotum in children: evaluation with color Doppler sonography AJR Am J Roentgenol 1992 158 613 617 1739005 10.2214/ajr.158.3.1739005

[B4] Chiang MC Chen HW Fu RH Lien R Wang TM Hsu JF Clinical features of testicular torsion and epididymo-orchitis in infants younger than 3 months J Pediatr Surg 2007 42 1574 1577 10.1016/j.jpedsurg.2007.04.020 17848251

